# There's no such thing as 'the economy', stupid: using Utopia to imagine society 'after money'.

**DOI:** 10.1007/s43253-023-00096-9

**Published:** 2023-04-14

**Authors:** Ruth Levitas

**Affiliations:** University of Bristol, Bristol, England UK

**Keywords:** Utopia, Sociology, Modern Monetary Theory, Unpaid work, Capitalism, Growth, 40, Climate change and Innovation

## Abstract

This paper uses qualitative sociological modelling to assess the transformative potential of Modern Monetary Theory as set out in recent work by Stephanie Kelton and Mariana Mazzucato. The starting point is Ruth Levitas’s (2013) Utopia as Method. This defines and develops Utopia as an exploratory, speculative sociological method characterised by a holistic and ecologically embedded approach. This is then used to explore the potential of MMT for envisioning social transformation. The paper argues that the neglect of unpaid work and a commitment to economic growth undermines the radical potential of MMT, as does the abstraction of ‘the economy’ from a wider understanding of social structures and processes. It suggests also that the project to use MMT to reform capitalism is fundamentally flawed since there is a conflict between the compound growth on which capitalism depends and the environmental imperatives that both Kelton and Mazzucato acknowledge.

In April 2020, early in the first wave of the COVID-19 pandemic, Arundhati Roy suggested it had exposed the fault lines of the capitalist economy and, in so doing, had allowed a vision of a better society, a potential new normal, to emerge: ‘Historically, pandemics have forced humans to break with the past and imagine their world anew. This one is no different. It is a portal, a gateway between one world and the next.’ (Roy 2020). By early 2021, at the peak of the second wave, glimpses of a qualitatively different and better way of living had given way to a race to vaccinate populations, at least in more affluent countries. By late 2022, although the pandemic was still killing millions across the world, concern had waned, and with it any suggestion that the pandemic itself would open the way to social reform. Nevertheless, hopes of social change were not extinguished. In this paper, I concentrate on two things. One is the possibility of a postcapitalist society, briefly glimpsed through the portal of the pandemic but elaborated through utopian thinking. The other is how far contemporary economics, and specifically Modern Monetary Theory, can be pushed in this direction using the work of Stephanie Kelton and Mariana Mazzucato.

Why Utopia? Utopia must be understood not as a blueprint or plan but as a method of exploring potential alternative futures. My work as a sociologist has been in three main areas: politics and social policy, with a focus on how discourses are deployed; poverty, inequality, and social exclusion, where I have been part of a team running nationwide surveys in the UK; and utopian studies. Levitas (2013) explores the connection between sociology and Utopia, suggesting that Utopia as method is a speculative sociology of the future. The distinguishing characteristic of sociology lies in looking at the social system as a whole—or, rather, imagining society as an integrated, if sometimes conflictual system. The models sociologists and social actors have of how society works are necessarily imaginary—as are the models economists and economic actors have, as Jens Becker (2016) has shown. They may correspond more or less accurately to the real world, but they nevertheless exist only as imaginary constructs. Sociological models are also necessarily qualitative. While some relationships between particular characteristics are susceptible to quantitative modelling and calculation, such as the cross-cultural relationship between mortality rates and inequality or between age and healthy life expectancy, the overall system is too complex for computer modelling. This is particularly the case because human behaviour and motivation are both product and producer of social processes and structures. Utopia as a method entails imagining society as it might exist rather than as it does. It has three aspects. In its archaeological mode, it means examining the models of society and social process and the characteristics of social actors underpinning claims about the social world, whether by politicians, economists, sociologists or others, and subjecting these to rigorous critique. In its architectural mode, it means imagining how the world might be otherwise. Clearly, this too must be subject to the same ongoing critique, so these two aspects are complementary. Essential to both is the third, ontological, mode, which asks what kind of people are imagined to inhabit this or an alternative world. In economics and in rational choice theory, the model of persons is generally rational economic man. However, half the population are not men, and people in general are neither rational nor do they often have a great deal of choice. The kinds of people we imagine emerging in interaction with new social institutions are an important element of imagining a new society. The merits of using Utopia to think about the future are twofold. First, it demands that we look at the social system and ideally its ecological embedding as a system and as a whole. Second, allowing ourselves to imagine a radical break from the present brackets the question of transition, of how (if at all) we might get there from here.

The demand of holism immediately takes us to the title of my paper. Looking at society as a whole, both as it is and as it might be, demonstrates that there is no such thing as ‘the economy’, but rather a whole system of practices and institutions that can be looked at from an ‘economic’ point of view, or from the perspective of processes of production, distribution, and consumption. This is more than an obvious point that ‘the economy’ is an abstraction. In the UK, we were told that ‘the economy’ fell by 50% during the first lockdown in 2020. It did not. It is getting on for 35 years since Marilyn Waring (1988) wrote If Women Counted, arguing for the consideration of nonmarket work in national accounting. It is over 25 years since Miriam Glucksmann (1995) argued that we must think in terms of the Total Social Organisation of Labour, or TSOL. Crude attempts have been made to quantify the contribution of unpaid work to total ‘output’. They are crude because there are hard questions about what constitutes work and because such calculations cost nonmarket behaviours at the price they might command in the market, and as we know, caring work and work by women are systematically undervalued by the market. But even so, they would effectively double the value of GDP. Hence, ‘the economy’ fell by at most 25%. And not even that, for what the TSOL reveals, and what Waring was alert to, is that activities can move across the market/nonmarket boundary without any change in the actual level of ‘work’ done. Quite clearly, a great deal of work moved back across the market/nonmarket boundary during the first lockdown. Childcare and home schooling are obvious ones. Others include domestic cleaning and cooking, and in my own case growing vegetables and baking bread. During the pandemic, hospitals in the UK were so overwhelmed that there were proposals to discharge patients early to be cared for at home where possible (or even where impossible), moving nursing and caring into the domestic, nonmarket arena. Women working from home had to juggle new domestic obligations to a greater extent than men. Among male academics, the rate of journal submissions went up during the Covid crisis, while for women, it went down.

Professional economists and sociologists are familiar with the shortcomings of GDP as a measure of productivity or prosperity, but the wider public is not. Consequently, one of the ‘revelations’ of the first lockdown was that those working in the genuinely essential services, the really socially necessary labour, such as refuse collectors, supermarket workers, delivery workers, care home staff, nurses and so on, are badly paid and often on insecure contracts. We know that GDP includes activities that are directly damaging to people and the planet, as well as many that are largely pointless. More than a decade ago, the New Economics Foundation tried to calculate the social value of a range of occupations compared to their market price and negative side effects (Lawlor et al. 2009). They estimated that in the UK, low-paid jobs such as childcare, hospital cleaning, and waste recycling generate between £7 and £12 in social value for every pound spent on wages; conversely, city bankers, advertising executives, and tax accountants destroy £7, £11, and £47 of social value respectively for every pound they create.

The pandemic very quickly exposed these and other issues, including the dependence of ‘the economy’ on unnecessary spending by over-indebted people. For some, principally, of course, the more privileged who could work from home and who lived in houses with gardens near pleasant open space, lockdown also offered glimpses of an alternative way of life. During the first wave, Martin Parker wrote:

Locked down, locked in, locked out, many people have had time to draw breath and notice some things about the way they live their lives. …[M]any of these changes have been positive. We will have driven and flown less, bought less, walked more, cycled more and cooked at home more. The air is cleaner and our cities are quieter. We can hear the birds, and the sky is not slashed by contrails. We might have done complex meetings and projects virtually, such as this book [and this conference], produced without a single face-to-face meeting. We might have spent more time with our families or housemates, helped neighbours or joined some mutual support or volunteering group. Perhaps we will have done some more gardening, crafts or art, or caught up on decorating or DIY. We might have read a book that we always wanted to read, or seen a film that really made us think, and that we wouldn’t have bothered with in busier times. We will have wondered what we really need and remembered to value some very ordinary things that we miss (Parker 2020:5).

Parker’s point was emphatically not that the lockdown was a jolly good thing, for he was very aware of the variation in experience by class, race, gender, and nation, as well as the devastation for those whom Covid meant they were literally unable to draw breath and for their families. It is that the glimpses of a different way of being, along with the exposure to dreadful inequalities of circumstance in life and risk of death, drew our attention to the world we have made and might perhaps remake.

Towards the end of the 1914–1918 War and the 1918–1919 influenza pandemic there was a discourse of catastrophe as opportunity. Roy and Parker’s responses mirror this. I salute their optimism, but the sceptic in me asks, opportunity for whom? The recovery from the 2008 financial crash resulted in the very rich becoming even richer. In Britain, and possibly elsewhere, the pandemic was an opportunity for profiteering aided and abetted by government corruption. It was, therefore, another opportunity for disaster capitalism as outlined by Naomi Klein (2007) becoming an excuse for greater austerity and depredations on the meagre resources of the poorer sections of the population. The prospect of mass vaccination closed over the rupture in the social imaginary, slamming shut Roy’s portal to a ‘new normal’, while we were warned that the minimal social protections put in place over the first year would have to be ‘paid for’ in the future. The challenge remains: how to imagine what would be entailed in developing those glimpses of another way of living into a potential alternative social system. And this is not just because of the social divisions underscored by the pandemic, inequalities Thomas Piketty (2014) demonstrates to be an inherent tendency of capitalism. The climate emergency itself demands such a response. Parker saw the response to Covid as a kind of dress rehearsal for that larger crisis, which was not even being addressed as a crisis because its effects were not yet widely experienced as palpably immediate. A mere 2 years later, that may be changing. 2022 brought episodes of searing heat in Europe and elsewhere, huge outbreaks of wildfires, and catastrophic floods in Pakistan. War in Ukraine following Russian invasion brought European sanctions against the aggressor and retaliation in the form of restrictions on gas supplies. The resulting energy crisis promised a bitter winter to follow the blistering summer and more reasons to reconsider the sustainability and equity of current practice. If David Harvey (2010) is right that capitalism depends on 3% compound growth, a sustainable society must be a postcapitalist one. As David Attenborough said in a 2013 speech to the Royal Geographical Society, anyone who thinks infinite growth is possible on a finite planet is either mad or an economist. Tim Jackson (2017, 2021) and others look, therefore, for prosperity without growth. There are questions about whether some sorts of growth are compatible with sustainability; if they are, it is not growth as conventionally understood.

Utopia as Method (Levitas 2013) outlines the principles of a sustainable, equitable postcapitalist society. It is not necessarily a society ‘after money’. That title calls for us to think about what money is, or perhaps more accurately, what money does. It may be a medium of exchange; a medium of communication through the price mechanism; a means of rationing; a work incentive; a vehicle of accumulation. In a postcapitalist society, there may perhaps be a call for something as a medium of exchange, but it will be a society in which money is not an instrument of power or a measure of capital accumulation. And there are also currencies that are not exactly money: Robert Owen’s labour notes in the nineteenth century, an early attempt at time banking; local currencies; local exchange trading schemes and time banks; and proposals for carbon currencies and care currencies.

The abolition of money and indeed property has long been a theme of literary utopias. Three of the best-known are Thomas More’s eponymous Utopia (1989 [1516]), Edward Bellamy’s Looking Backward 2000–1887 (2003[1888]) and William Morris’s News from Nowhere (1902[1891]). It is More, of course, who gave us the very word utopia, itself a pun on eutopia (good place) and outopia (no place). The depths of More’s critique of capitalism are apparent in his own preface to his outline of a different society. Book One is largely about the merits of accepting appointment as adviser to a king (which may also have some contemporary relevance). But it also includes an excoriating critique of his own time, especially the social consequences of enclosures as people were driven off the land to rear increasing numbers of sheep for their valuable wool, and thus plunged into poverty, starvation and crime, and hung for theft. As the political economist Ellen Meiksins Wood (1999) argued, this moment of enclosure that More confronts is precisely the founding moment of capitalism, as driving people from the land creates the landless workforce compelled to sell their labour power. Both William Morris, who produced an edition of Utopia at his Kelmscott Press and wrote a preface to it in 1893 (Morris 1893) and the Marxist critic Fredric Jameson (2005) writing more than a century later, hailed the abolition of money and property as More’s key invention. If we look closely, though, there is rationing. Although households may take what they need from the collective storehouses, it is only heads of households (all men) who can authorise this.

Nearly 400 years after More, Edward Bellamy’s Looking Backward also attempted the abolition of money and explicitly of financial services. At one point, the main protagonist, Julian West, wonders, ‘What have you done with the … bankers? Hung them all, perhaps …?’ (Bellamy 2003: 93). Well, no. They have merely been abolished. West’s informant observes that those who think the financial sector is the core of the economy mistake ‘the throbbing of an abscess for the beating of the heart’ (Bellamy 2003: 234). In twenty-first-century Boston, money is not used as a medium of exchange because no such exchanges are imagined among the general population. Economic equality is imposed, for all citizens (men and women) receive a statutory allocation against which they can draw for goods or services. So buying and selling do indeed take place, although the currency is virtual. The credits are said to be set at an ample level and very rarely exhausted. They cannot be carried over from year to year, so cannot be used for saving or accumulation. Yet if they do not act as a constraint on consumption, as a rationing device, I wonder why they are necessary at all.

Morris’s News from Nowhere was, as is well known, written as a riposte to Bellamy’s state socialism. Morris’s utopia does indeed abolish both money and rationing. Markets are merely points for the collection and distribution of goods. Where both More and Bellamy have systems of compulsory labour incentivised in various ways, Morris does not. In his review of Bellamy, Morris insists that ‘the true incentive to useful and happy labour is and must be pleasure in the work itself’ (Morris 1889: 506). Morris’s most utopian leap is the abolition of any calculus or rationing. Just as in the post-war years social democracy allowed affluent countries to treat clean water, education and healthcare as goods freely available to all, a Morrissian perspective sees no logical reason why such open access should not apply also to food, energy, or transport in a future where ‘[w]e shall no longer be hurried and driven by the fear of starvation’ (Morris 1884). Or, as the President of Ireland, Michael D. Higgins, wrote. ‘In a republic, the right to shelter, food security, education, a good environment and freedom from fear and insecurity from childhood to old age, must be the benchmarks’ (Higgins 2011: 61). A form of society built on the principle of solidarity stands as an alternative to the abstract entity of the markets.

George Osborne, architect of Britain’s policy of austerity after the banking crash, argued that we need to do more and we need to do it faster. Quite the opposite, Levitas wrote: ‘We need to do less, and we need to do it more slowly. Doing a lot more nothing, including sleeping, would reduce resource consumption, lower stress levels and enable social relations more conducive to dignity and grace’ (Levitas 2013: 203). This resonates with Covidtopia’s appreciation of slowing down. I argued that the goals of human dignity and human flourishing depend on much greater equality: ‘The good society has equality at its core. It demands the public ownership and control of assets currently in private hands. … The forms of work generated by capitalism do not cultivate craftsmanship in the deepest sense. A radically different form of economy and society oriented to human need rather than profit is the starting point for fuller, freer, more satisfying human relationships’ (Levitas 2013: 215). The principles of this society are greater equality; basic income and high levels of public services; good work; a radical revaluation of care; and an ethic of care that extends to care for the environment.

During the pandemic, basic income went from being the aspiration of a few marginal eccentrics to a mainstream aspiration, albeit in many cases as an emergency substitute for an overwhelmed benefit system. In terms of a society ‘after money’ it is of course a transitional demand; money will still serve as a medium of rationing as in Bellamy, rather than all goods and services being freely and universally available as in Morris. But most calls for UBI were for an emergency measure for income support through the pandemic, and where UBI is advocated as a substitute for social security schemes, it is generally expected to be set at a low level. Most BI experiments are of this kind. Slightly more radical is the policy being developed by the pressure group Compass in Britain, which would introduce BI alongside the current benefits system, with an allowance of £60 per week for each adult and £40 for each child. Stewart Lansley, who has modelled this, argues that this could be funded by the abolition of the tax-free personal allowance, would be largely revenue-neutral, and would also be radically redistributive. The top 20% of the population would experience a small net loss in income, but the poorest 30% would gain substantially (Lansley and Reed 2019). Though this might be very successful as a springboard out of poverty, it is very different from a scaled-up version of BI set at a higher level—say, average income—across the population, together with generous allowances for children. A higher level of BI would eliminate the huge hidden costs of child poverty, which would be abolished at a stroke, and (again, in a transitional state), the rates of taxation of earned income above this social floor could be adjusted accordingly. Lansley and Reed explicitly reject this as too expensive and as utopian, seeing it as more important to establish the principle of universality in the short term—and this may indeed be an appropriate first step. In the longer run, however, the more utopian approach can help us to think more radically about the future. Another objection to BI sometimes raised is that its very unconditionality enables people to ‘do nothing’. In my view, enabling people to do nothing is part of the point. It enables that slowing down noted as a positive experience of the pandemic for those in privileged positions. It would improve working conditions at a stroke, as no one would be compelled to put up with extreme tedium or exploitation. And in reality, the so-called ‘nothing’ that the so-called ‘economically inactive’ do is rarely nothing. It includes all kinds of activities, perhaps designated as unpaid work, perhaps as leisure or hobbies, such as cultivating allotments, art, music, and, above all, caring.

Putting care at the centre of a new society has been advocated by many, notably in the attempt to substitute a care ethic for a work ethic. It requires a complete revaluation of care and its role in social life. If we consider what would be entailed in the abolition, rather than simply the alleviation, of child poverty, we find everything would need to change (Levitas 2012). The same is true of putting care at the centre of social concerns. For then we recognise that care is not simply a residual category, the business of parents when their children are young, and an unfortunate necessity for the frail elderly and people with disabilities. We all need care. This ‘work’ of caring is what Marxist feminists in the 1970s and 1980s called the extended reproduction of labour power—all those services that enable us to get up and go to work the next day, whether it be food preparation, cleaning, washing, nurturing, and what is now termed ‘self-care’. If we turn it around and make that, rather than the ‘getting up and going to work’ the centre, life would look very different.

There is a vast literature here, and I make no attempt at an overview. Two recent publications, however, approach these issues in different ways. Madeleine Bunting’s (2020) Labours of Love combines an analysis of the crisis in care in Britain, ranging from childcare to domiciliary and residential care of the elderly, with an ethnographic study combining observation and interviews with carers. She also ends with a chapter on possible futures, including the use of AI and robots, which, as she says, is potentially dehumanising. Bunting also discusses more favourably a system in Japan where you can earn credits by caring for unrelated elders in your own district that can be used to purchase care for your parents if they live elsewhere. But this is simply a form of time banking, not the basis of a transformation of society. The Care Manifesto (The Care Collective 2020), on the other hand, does proceed from the universal necessity of care to a completely restructured political economy at both state and supra-state levels. It also extends the notion of ‘care’ to care for our home, the planet on which we live, and therefore care for the environment.

The ways that we live need to change. This means a change in the ‘structure of the economy’, or put another way, in our means of livelihood and ways of life. To reiterate: this is not a blueprint or a plan, but nor is it a fantasy or mere wish fulfilment of a nonexistent place whose realisation is impossible or even dangerous. It is a provisional exploration of a potential alternative future. And for those who wish to dismiss it as unrealistic, what is truly unrealistic is to imagine that we can continue as we are. It is necessarily provisional, for although one of the strengths of utopian thinking is that it permits a radical break from the present, that break is always incomplete. As we imagine Utopia, we are always conditioned by our preoccupation with the present, and with the conditions we seek to critique or change; and we are also limited by what it is possible for us to imagine given our own historical and social location. And if the radical break is a stimulus to imagining the world otherwise, we do also need to return to the question of transition. Difficult questions about political agency arise here, which are beyond the scope of this paper. I would only stress the importance, as Pope Francis says, of collective aspiration here, of dreaming together. But besides the very real difficulties of wresting control of the world’s wealth from the tiny minority who currently control it, we need to change ways of thinking about the society and ‘the economy’ that we currently inhabit.

I explore from a sociological perspective how far Modern Monetary Theory and critical economics might take us in seeing the world sufficiently differently for this radical utopia to seem less unrealistic. Stephanie Kelton (2020) and Mariana Mazzucato (2019, 2021) are both ‘insider economists’, in that they are advisers to senior politicians and governments. Both (and perhaps especially Mazzucato) are clearly trying to redefine economic thinking in a way that facilitates radical social change. Ultimately, I am critical of both for focusing on ‘the economy’ rather than the total social organisation of labour and consequently being insufficiently holistic and insufficiently utopian.

Kelton has been described as a ‘prophetic economist’. She was the chief economist on the US Senate Budget Committee (minority staff), as well as an advisor to Bernie Sanders’ campaign in 2016. The Deficit Myth (Kelton 2020) should be required reading for all political commentators. We are regularly told that the huge economic deficits incurred in countering the economic impacts of the pandemic and indeed the energy crisis will have to be ‘paid back’. Kelton’s central point is that this is not true and that the error arises from looking at national budgets as if they are analogous to household budgets.

She concedes that currency users such as households, companies, local authorities, and individual countries within the Euro can run out of money. But currency issuers cannot. They could when currencies were tied to the gold standard, but this has not been the case since the 1970s—that is, for the last half-century. The problem for Greece is that using the Euro rather than the Drachma meant it was a currency user. But currencies issued by national governments are ‘fiat currencies’, and these economies are nothing like household budgets as governments can simply issue more money. This is what is meant by quantitative easing. There are limits to printing money (or notionally crediting it to computerised accounts, since much money never physically exists), but they have to do with the potential risk of inflation, not with the size of the deficit. Inflation is assumed to be a danger if unemployment falls too low, but no one knows what constitutes too low, so they guess and mostly guess wrong.

Kelton talks about the analogy with households as a ‘myth’. I would call it ideology—an account of the world that operates to disguise what is really going on. The deficit is mostly used as a justification for austerity, and therefore there is a political interest in this misunderstanding. Kelton also draws attention to the massive levels of inequality, arguing that living standards have barely risen in the last 4 [or 5] decades because of the sequestration of assets by the rich, a process that damages the economy because it reduces both investment and spending power (see also Lansley 2012). To counter unemployment, the state should become the employer of last resort, with a living wage payable for socially useful work in projects organised and chosen at local or community level—that is, centralised funding with decentralised control and decision-making. Then people will be able to do useful things, learn new skills, and maintain their employability until private sector opportunities pick up.

Kelton also addresses the widely held assumption that governments need the receipts of taxation to fund spending. Taxation, she argues, does not exist for this purpose and certainly does not need to precede government spending. The function of taxation is two-fold. First, it is redistributive. The highest rate of tax in the USA was once 91%, just as it once was in the UK. Such high rates are historical, but Leonard Trelawney Hobhouse, the first professor of sociology at the London School of Economics, suggested a century ago that 80% of all value is socially created, so an 80% top rate of tax would be congruent with this, especially in the light of steeply rising ratios of CEO pay to that of ordinary workers. It would also be congruent with new arguments for maximum earnings or incomes alongside minimum incomes, called ‘limitarianism’ (Robeyns 2017, 2019, and 2022). The second function of taxation, according to Kelton, is as a work incentive: people need to earn currency with which to pay taxes—though, as noted above, Morris argued that the true incentive to useful and happy labour is and must be pleasure in the work itself. In her last chapter, ‘Towards a better economy’, Kelton says that the question of whether a programme can be afforded needs to be thought about not in terms of money and deficits but in terms of the real resources deployed—resources of materials, skills, and people.

This argument is critically important in unravelling the ideological framing of what is and is not possible in ways that are quite congruent with the provisional utopia or transitional demands set out above and can be pushed quite a long way. Her local job creation schemes could be radical, although that would depend on the actual levels of local control consistent with securing central funding. But there are limits that derive from thinking about ‘the economy’ rather than society more broadly. Kelton’s notion of a healthy economy is conventional. She may not make economic growth central to her argument, but nor does she make a case against it or argue strongly for sustainability. And there is no mention of unpaid work (other than in the critique of GDP), so the notion of what constitutes ‘the economy’ remains intact, and the question of what constitutes ‘socially useful work’ is uninterrogated.

Both Kelton and Mazzucato identify the 1970s as an important breakpoint in economic reality and accounting. For Kelton, the abolition of the gold standard in that decade makes talk of the deficit a myth. For Mazzucato (2019), the 1970s saw the beginning of accelerating financialisation as the financial sector managed to establish the definition of their activities as productive, causing a swing from value determining price to price being taken as the measure of value. This led to a huge rise and overvaluation of the financial sector, which meant that grossly overpaid people in that sector could claim credit for economic growth. Linked to this was a process of accelerating inequality, continuing now into its fifth decade. In The Value of Everything, Mazzucato explores changing definitions of value and of productive and unproductive labour, argued through the history of political economy and latter-day pseudo-scientific economics. She is excoriating about conventional economics based on marginal utility, as opposed to political economy, stating that ‘economics emerged as a discipline in large part to assert the productive primacy of the private sector’ (2019: 239). Her critique of national accounting and GDP includes its neglect of unpaid work, its inclusion of negative effects, the overvaluation of the financial sector, as well as the inflation of GDP by the inclusion of imputed rents and a consistent undervaluing of the productive role of the state. She also documents the rise in inequality since the 1970s in parallel with this financialisation, the sequestration of wealth and income by a tiny minority (especially the top 0.1%), the falling share of income going to labour rather than capital, the fall in real wages, and the rise in household debt. Hers is a detailed analysis of the processes of financialisation and their consequences, and the transformation of public goods into private goods. At the core of her argument is that the creation of value is collective and needs to be at the centre of economic reasoning and that public investment is critical to the nature and direction of economic growth.

There is much here to agree with and much to appreciate in the clarity of the argument. Mazzucato’s purpose—like my own—is ‘a better future for all’; to ‘dream of a better future and try to make it happen’ (2019: 280). She aspires to an economy with ‘more fulfilling jobs, less pollution, better care, more equal pay’ (2019: 279); a stakeholder rather than shareholder capitalism; a new discourse on public value and the common good; new metrics. Yet there are only a few passing references to the climate emergency and occasional references to some rather vague wider ‘social’ that exists alongside the economy and government. Like Polanyi, she accepts that ‘markets are deeply embedded in social and political institutions’ (2019: 275). Therefore, she argues, we can reshape markets to ‘produce desirable outcomes such as green growth’ or a more ‘caring’ society with care influencing the type of social and physical infrastructure that is built (2019: 275), although the passive voice here suggests uncertainty about agency. The stakeholding approach implies that companies are ‘social entities’ (2019: 268). Innovation needs to be reshaped ‘to battle societal and technological grand challenges—like climate change or social care’ and ‘we can choose and direct green and care as the new paths for innovation’ (2019: 226). The challenge to repair damage to the environment ‘requires a societal commitment to new, less physically materialist approaches to the way we live’ and ‘a green revolution’ (2019: 279). It is perhaps this extreme vagueness about the society that explains why, despite noting that unpaid work does not figure in GDP, it does not figure in her envisioned better world either.

Mazzucato’s 2021 book, Mission Economy: A Moonshot Guide to Changing Capitalism, develops some of the themes of her earlier work. The case for radical change is overwhelming, and change must be led by governments because ‘markets will not find a green direction on their own’ (2021: 143). The challenge is to transform capitalism, which requires ‘restructuring business so that private profits are reinvested back into the economy rather than being used for short-term financialized purposes’ (2021: 208). Over and over, this means ‘rethinking’ capitalism, ‘restructuring’ capitalism, ‘doing capitalism differently’ (2021: 209), and addressing our ‘dysfunctional form of capitalism’ (2021: 15). Much of this explicitly refers to the debates about stakeholding that were prevalent in the 1990s (see Levitas 2005). The issues she identifies are short-termism, financialisation, weak government, and the climate emergency. There are references to ‘institutions’ and society and culture. She sees her project as ‘imagining a better future’ and claims the crisis as an opportunity: ‘a time of crisis is exactly the moment to reimagine what type of society we want to build’ (Mazzucato 2021: 8). Indeed, she ends with the quote from Arundhati Roy with which I opened this paper (Mazzucato 2021: 212).

In looking at new theory, Mazzucato herself cites several women scholars who, as she puts it, put life at the centre of the economy, not the economy at the centre of life. Except that they do not, and she does not. There is no mention of unpaid work, despite a discussion of the sustainable development goals. SDG 5 on gender equality has six targets, the fourth of which is to ‘recognise and value unpaid care and domestic work through the provision of public services, infrastructure, and social protection policies’ (2021: 110). Watching Mazzucato and Diane Coyle giving evidence to the British Parliamentary Committee BEIS (Business, Energy, and Industrial Strategy Committee), it is notable that neither of them raised any question mark over economic growth or how we measure it. And Mazzucato, like Kelton, is absolutely committed to economic growth. For her, ‘the real question is how government spending and investment can create long-term growth’, even if the direction is more important than the rate of growth. This is consistent with her project to mend rather than end capitalism. David Harvey’s analysis should make us sceptical about whether capitalism itself is compatible with environmental limits.

The strands of critical economics represented by Kelton and Mazzucato go a long way to break down the ideological justifications of predatory capitalism. But they do not go far enough. They focus on ‘the economy’ rather than taking a more holistic view of a good society, our means of livelihood and means of life, and our embeddedness in the living planet that we are killing inexorably. This triad of economy, state, and society misses a large part of the total social organisation of labour. Hence, my title: ‘There is no such thing as the economy, stupid’. Kelton and Mazzucato are very far from being stupid. They are radical—visionary even—but blinkered by the very discipline they criticise. To go further, their analysis needs to be more sociological, more holistic, and more radical—yes, more utopian. We need the more holistic visions of utopian sociology to secure our future, ‘In this very world, which is the world of all of us, the place where, in the end, we find our happiness, or not at all’ (Wordsworth 1805).
